# Changing the trajectories of mental health difficulties in Norfolk and Suffolk: a research-priority-setting project with patients, the public, clinicians, policymakers and other stakeholders—study protocol

**DOI:** 10.1136/bmjopen-2024-093980

**Published:** 2025-01-04

**Authors:** Sherifat Oduola, Craig Morgan, Jayati Das-Munshi, Niall Broomfield, Helen Parretti, Kristy Sanderson, Caitlin Notley, Amy Zile, Sol Morrissey, Zarnie Khadjesari, Joni Holmes

**Affiliations:** 1 School of Health Sciences, University of East Anglia, Norwich, UK; 2 Norwich Epidemiology Centre, University of East Anglia, Norwich, UK; 3 Health Service and Population Research Department, Institute of Psychiatry Psychology and Neuroscience, London, UK; 4 ESRC Centre for Society and Mental Health, King's College London, London, UK; 5 Psychological Medicine, King's College London Institute of Psychiatry Psychology and Neuroscience, London, UK; 6 Norwich Medical School, University of East Anglia, Norwich, UK; 7 National Institute for Health and Care Research Applied Research Collaboration East of England, University of East Anglia Faculty of Medicine and Health Sciences, Norwich, UK; 8 School of Psychology, University of East Anglia, Norwich, UK; 9 MRC Cognition & Brain Sciences Unit, University of Cambridge, Cambridge, UK

**Keywords:** MENTAL HEALTH, Caregivers, Delphi Technique, Awareness

## Abstract

**Abstract:**

**Introduction:**

Mental health problems are the most significant cause of disability and have high annual economic costs; hence, they are a priority for the government, service providers and policymakers. Consisting of largely coastal and rural communities, the populations of Norfolk and Suffolk, UK, have elevated burdens of mental health problems, areas with high levels of deprivation and an increasing migrant population. However, these communities are underserved by research and areas with the greatest mental health needs are not represented or engaged in research. This National Institute of Health and Care Research-funded project aims to bring together key stakeholders to conduct extensive scoping work to identify mental health needs and priorities as a basis for conducting larger research to address the identified priorities over the next 5 years.

**Methods and analysis:**

This 12-month mixed-methods research-priority-setting project consists of five phases. It is being conducted in Norfolk and Suffolk counties in the East of England, UK. Underpinned by Delphi methodology, it will adopt the James Lind Alliance approach to identify priorities for mental health research for the populations of Norfolk and Suffolk. The project will use multiple methods, including mapping and identification of stakeholders, online questionnaires, face-to-face focus groups and interviews, and consensus meetings with experts and mental health stakeholders. Key evidence-informed priorities will be collaboratively ranked and documented, and a final top 10 research priorities will be identified to inform future research, policy and service provision.

**Ethics and dissemination:**

This study was approved by the University of East Anglia’s Faculty of Medicine and Health Research Ethics Committee (reference: ETH2324-2542), Norwich, UK. Research findings will be disseminated through workshops with stakeholders and collaborators and via peer-reviewed scientific publications, presentations at academic societies, blogs and social media.

**STRENGTHS AND LIMITATIONS OF THIS STUDY**

- This study will bring together key stakeholders including people with lived experience, mental health service providers, local authorities, policymakers, voluntary organisations, academics and members of the public to identify mental health research priorities specific to Norfolk and Suffolk populations.
- In-depth consultation and data collection with people with lived experience of mental health difficulties who are often under-represented in research, particularly in rural, coastal and migrant communities.
- The current study is limited to participants aged 16 and over. However, family carers or parents of children with mental health difficulties will be included. Nonetheless, future mental health research priority-setting involving children is warranted.

## Background

 Mental health problems are the most significant cause of disability,^1^ costing the NHS over £118 billion annually^2^; hence, they are a priority for the government, service providers and policymakers. The burden of mental illnesses such as anxiety, depression, schizophrenia, bipolar disorders, eating disorders, addictions, autism spectrum and personality disorders is considerable to individuals, families, the economy and wider society.^3 4^ There is good evidence that several environmental, social and psychological risk factors are linked to the onset, course and outcomes of these conditions, including area-level deprivation, fragmentation, migrant or ethnic minority status, socioeconomic disadvantage and childhood adversity.^5^,^8^ However, the distribution and effects of social risks vary significantly by geography, and regional disparities in access to care and outcomes exist.^9 10^ Much of our understanding of mental healthcare and outcomes has been gleaned from studies involving urban and city populations. This means we do not know enough about how rural or coastal living impacts the mental health of around 10 million people in rural areas (villages, hamlets, market towns) in the UK.^11^

Globally, mental health difficulties account for a high burden of disease among young people, with an estimated prevalence of 14%, although with substantial variation across countries and regions.^12^ In the UK, 17.4% of people aged 6–19 years had a diagnosable mental health disorder (eg, anxiety, depression) between 2020 and 2021,^13 14^ increasing to 20.3% in 2023.^15^ These difficulties account for three of the top six causes of disability-adjusted life years lost among people aged 10–24 years,^16^ and suicide is the third leading cause of death in this group.^17^ It is also well-documented that around 75% of all lifetime mental disorders occur before the age of 25.^17 18^ Therefore, considering the potential negative impact of mental illness on adult outcomes, adolescence is a critical period for early detection of mental health difficulties, rapid access to support and treatment and mental health promotion. This is especially important in rural areas with an increased risk of social isolation, stigma, limited infrastructure and poor access to care.^19^,^21^ Further, coastal communities face significant public health challenges, including a high burden of mental health difficulties and poor access to care, which are set to get worse without a vigorous and systematic approach to addressing the challenges.^22^ These challenges are driven largely by socioeconomic stagnation and environmental factors, including deprivation, physical isolation, deindustrialisation, transient populations and inward and outward migration of older and young people, respectively.^23 24^ The combination of these characteristics in coastal communities, including in our case, Great Yarmouth (Norfolk) and Lowestoft (Suffolk), increase the risk of mental health problems.^24^ Table 1 summarises the decline over time in the UK coastal communities.

**Table 1:** An overview of decline in UK coastal communities: a description of economic, social and health context

Period	Economic, social and health context
19th Century	Coastal areas thrive during the Victorian era, with the development of railways leading to increased tourism and the rise of the seaside holidays.^54^
20th Century	Seaside towns maintained their popularity in the early 20th Century, with a transition from health to entertainment, bringing new activities and people from different socioeconomic backgrounds to UK coastal towns.^55^ In the late 20th Century, competition from commercial air travel reduced visitor numbers to coastal towns, leading to economic downturns.^56^
21st Century	Regional disparities in digital connectivity, less reliable public transportation, deindustrialisation and insufficient infrastructure development have led to some coastal communities being ‘left behind’.^57^ In Norfolk and Suffolk, economic decline resulted from the contraction of the shipbuilding, fishing and tourism industries.^24^ Severe coastal erosion issues and extreme flooding risks posed by climate change exacerbate risks of social, health and well-being issues.^23^ England’s Chief Medical Officer calls for urgent actions to reduce health inequalities and improve health outcomes in coastal areas.^22^

### The Norfolk–Suffolk context

Consisting of coastal and rural areas, the populations of Norfolk and Suffolk have an elevated prevalence of mental health problems and areas of high levels of deprivation. Compared with England’s average of 22.6%, the prevalence of anxiety and depression in Norfolk and Suffolk is 25%. The rate of hospital admissions due to self-harm among people aged 20–24 years in Norfolk and Suffolk is 366.5/100 000 compared with 340.9/100 000 in England.^25^ Around 40% of people in some of our coastal and rural communities live in the most deprived areas in England, and the life expectancy gap between the most and least deprived areas is 7.4 years for men and 4.4 years for women.^26^

Between the 2011 and 2021 Censuses, the population rose by 5.4% in Norfolk and Suffolk and is projected to increase by approximately 6.7% through internal and international immigration by 2029.^26 27^ In Suffolk, the proportion of people from black African/Caribbean or mixed ethnic backgrounds increased by 48.4% from n=6854 in 2011 to n=10 168 in 2021.^27^ The link between migration and increased risk of mental health difficulties (such as anxiety, depression and psychosis) has been established.^28^,^30^ Therefore, the evidence base for the impact of immigration and the effect of rural and coastal living on mental health must be developed and would potentially inform efforts to reduce health inequalities.

There is a well-established link between physical and mental health,^31 32^ and there have been several calls for greater integration between the two, for example, *No Health without Mental Health*^33^ and *Supporting the physical health of people with severe mental illness*.^34^ Despite this recognition, progress is slow in narrowing the mortality gap. Premature deaths from severe mental illnesses such as schizophrenia have been linked to the coexistence of other long-term health conditions (eg, cardiovascular disease, diabetes, stroke), usually referred to as multimorbidity,^35^ but how and why these occur in and affect people living in rural/coastal areas is poorly understood. In Norfolk, for example, healthy life expectancy for women is lower, at 62.4 years, compared with 62.7 years in England, and it has decreased over the last few years. This means that the period females spend in ill health in Norfolk is getting longer.^26 36^

Addressing disparities in mental health research is a key government objective. However, for research to have a real impact and make a difference in people’s lives, it needs to be developed and prioritised with key stakeholders and those whose lives will be most affected by the research. Informed by a community-based participatory research approach,^37^ our partnership with those directly affected by and knowledgeable of the local issues that affect mental health means that our research has real potential to influence the implementation of evidence and evidence-based interventions into practice and policy.^38^ Furthermore, research needs to be inclusive to ensure it serves those who need it the most.^39^ Our research acknowledges the intersectionality theory which recognises everyone’s unique experience of discrimination and exclusion^40^,^42^; therefore, we pay careful attention to the intersecting characteristics of participants, such as age, sex, ethnicity and social circumstances. This is the key premise underpinning this Norfolk–Suffolk Mental Health Development Award (MHDA) project.

### Aim and strategic goals

Our vision is to establish the National Institute of Health and Care Research (NIHR) Mental Health Research Group (MHRG) at the University of East Anglia (in collaboration with the Centre for Society and Mental Health (CSMH), King’s College London), which aims to develop an applied mental health programme of work that examines how mental health problems emerge across the lifespan, how we can intervene quickly, prevent ill health, promote positive mental health and well-being and reduce the impact of deprivation on health in our communities. This is particularly important as most mental health research to date has been conducted in urban areas in the UK, neglecting rural and coastal communities like ours. To achieve this vision, we need to build partnerships, achieve local buy-in and work with communities to identify priorities through this initial project supported by the NIHR MHDA. The current project centres on understanding the unique needs of our communities and mental health research priorities by engaging key stakeholders, communities, local partners, and experts to gain long-term investment in joint working. We will address the following objectives:

1. Identify and bring together people with lived experience, mental health service providers, local authorities, policymakers, charities, schools/colleges and researchers in Norfolk and Suffolk.
2. Assess the relevance of the available research priorities using the James Lind Alliance ‘overarching priority topics’ for the health and care research toolkit to identify areas of need.
3. Work with stakeholders to produce a list of up to 10 applied mental health research priorities in Norfolk and Suffolk to inform the NIHR-MHRG research programme.
4. Translate the top 10 identified priorities into answerable research questions to be addressed by a larger MHRG programme.
5. Finalise and design MHRG research programmes to address the identified research priorities.

A larger goal is to build strong research collaborations with our local communities, Integrated Care Boards (ICBs), mental health providers, voluntary and non-academic organisations, as well as between the University of East Anglia (UEA) and CSMH King’s College London, by enabling cross-institution working, building research capacity, and training programmes and laying the foundation for long-term ground-breaking research to improve lives and promote well-being and economic growth in our local populations.

## Methods

### Study design

This project is underpinned by the Delphi methodology. A Delphi study technique provides a structured method to facilitate effective communication with stakeholders, leading to the development of consensus among panel members on a complex problem.^43^ It involves interactive discussion and assumes collective judgments are more valuable than individuals. A mixed methods design will include an online survey, stakeholder focus group (FG) discussions and prioritisation workshops.

### Study status

This project will begin recruitment in June 2024 and is expected to be completed by May 2025

### Study settings, participants and eligibility

All stakeholders of applied mental health research in Norfolk and Suffolk will be eligible to participate if they are 16 years and above and fluent in English language. This includes people with lived experience, mental health service providers, other health and social care professionals, local authorities, policymakers, charities, youth groups, educators, community groups and researchers. It is anticipated that all participants will be sampled from the communities and organisations in Norfolk and Suffolk. Everyone who meets the above criteria will be considered eligible to participate in the study, and no further exclusion criteria will be applied.

#### Procedure

Several methods have been published describing approaches for setting priorities for health research, but it has become apparent that there is no single best practice.^44 45^ Defined as an interpersonal framework to build consensus, the James Lind Alliance (JLA) framework is arguably the most used approach in setting health research priorities; it focuses specifically on the effects of treatment interventions and aims to generate a top 10 research priority list.^46^ We will identify existing mental health research priorities from the JLA ‘overarching priority topics’ for the health and care research toolkit^47^ to see whether they are relevant to populations in rural/deprived areas of Norfolk and Suffolk and whether there are unanswered questions/topics that, if examined by research, could make a real difference to people’s lives. Our priority-setting exercise will follow the JLA process and guidance across five phases as follows:

1. Identifying and mapping stakeholders
2. Consultation
3. Collation (data analysis)
4. Prioritisation
5. Research programme development

### Phase 1: identifying stakeholders (study objective 1)

*Aim:* This initial phase aims to identify all key regional stakeholders in mental health, including service users, clinicians, third-sector organisations, commissioners, policymakers, social care professionals, educators and local government.

*Procedure:* We will identify participants through stakeholder mapping to obtain a broad range of respondents from different demographic and professional groups. We will use various sources to map and identify the stakeholders, including existing research databases, personal contacts, online searches and partner organisations; this will be an iterative process. We will identify the stakeholders’ expertise by grouping them into expert pools: Expert pool 1: people with lived experience of mental health difficulties or family carers or members of the public; Expert pool 2: mental health clinicians and other health and social care professionals; Expert pool 3: third sector organisations and community groups; Expert pool 4: researchers/academics; Expert pool 5: local authority/schools, employers; Expert pool 6: policymakers, that is, ICB. The mapping of stakeholders will give in-depth insights into the structures and operation of mental health, education, government systems, and community groups across Norfolk and Suffolk.

### Phase 2a: consultation—online survey (study objective 2)

*Aim:* The consultation phase aims to gather stakeholders’ perceptions on the available mental health research priority topics and their ability to identify unmet mental health needs and research priorities.

#### Participants and sampling

Participants will include the expert pools identified in Phase 1. This will include people with lived experience and members of the third-sector organisations involved in mental and public health decision-making at macro, meso or micro levels across the integrated care systems. We are mindful of the difficulties in involving under-represented groups in research, particularly where they also have mental health difficulties. For this reason, we will conduct in-person consultation and FG discussions (or interviews if preferred) with under-represented groups at accessible locations, as set out in Phase 2b. Collecting data using different formats (ie, online and in-person discussion) has been used in previous studies and is in keeping with the JLA guidelines.^48^ All eligible participants will be informed about the project via our partner organisations, which will act as gatekeepers. We will also promote the research via social media, our networks, partner organisation websites, newsletters and posters.

#### Data collection

In collaboration with our project Steering Group (SG) and informed by the JLA ‘overarching priority’ topics for the health and care research toolkit,^47^ we will develop an online questionnaire using Microsoft Forms to capture participants’ views of areas of need for mental health research in Norfolk and Suffolk. From the JLA ‘overarching priority topics’ toolkit, we will identify mental health-related questions/topics and create a list of priorities under the following domains: children and young people’s mental health; the link between physical and mental health; the impact of rural and coastal living on mental health; access to mental healthcare; migration and mental health; social and health inequalities; mental health promotion and prevention. The survey questionnaire, including the project information sheet, will be sent to all eligible participants via our partner organisations, asking them to be gatekeepers and share the survey with their teams and service users. Participants will be asked to rank their priorities from the list of research statements provided on a 3-point Likert Scale (0=low priority; 1=moderate priority; 2=high priority), as recommended by JLA.^46^ To identify any unanswered research priorities, all participants will be asked to list three mental health research priorities that were not included in the predefined priority list, and they will be asked to rank their identified priorities on a 3-point Likert Scale as above. We will adapt the questions by the expert pool. Participants identifying as someone having lived experience of mental health difficulties will be asked about their diagnosis, duration of illness and whether they have had access to care/treatment. They will be asked about the potential influence of the social environment on their health. The survey will be anonymous, and a consent statement will be included in the questionnaire (see online supplemental material 1). All participants will be asked for basic demographic details, for example, age, gender and ethnicity, which will enable us to keep track of the diversity in the sample. If recruitment is low from a particular group, we will adapt our recruitment strategy to target such groups.

The survey data collection will be open for 3 months to allow participants sufficient time to decide whether they want to participate. To increase the likelihood of reaching all potential participants, we will ask each participant to nominate another individual or organisation involved in mental health service use, provision and decision-making. An optional prize draw of twenty £25 Amazon vouchers will be offered. As the survey is anonymous, participants will be asked to provide an email address if they wish to be entered into the prize draw. We will also ask participants to indicate their willingness to participate in the subsequent research prioritisation workshops.

#### Sample size

The JLA does not recommend a minimum or maximum number of responses.^49^ However, we will aim for 165 responses, indicating an average response rate based on previous JLA priority-setting exercises with rapid approaches.^48^

### Phase 2b: consultation—in-person survey completion and FG discussions (study objective 2)

*Aim:* Phase 2b aims to purposively seek the views of people with lived experience, including family carers and those often under-represented in research on unmet mental health needs and identify research priorities using the questionnaire from Phase 2a.

#### Participants and sampling

Participants will include marginalised groups (as described above). Members of our SG and Lived Experience Advisory Group (LEAG) will facilitate the recruitment of participants from their respective organisations or community groups and will act as gatekeepers. Participants will not need to complete the survey (2a) to participate in FG discussions/interviews (2b).

#### Data collection

Facilitated by a research associate and LEAG lead, we will engage participants in FG discussions or 1:1 interviews, if preferred. The FG discussion will begin by asking the participants broad questions about current gaps in mental healthcare. Then, participants will be asked to complete a consent form and the survey questionnaire (if they wish, paper copies and/or an electronic device will be provided); the researcher will support them if required. Next, as recommended by the JLA,^46^ we will explore questions in the survey, as appropriate, to gain a deeper understanding of any unanswered questions. To ensure all potential participant groups get a chance to contribute to the study, meetings and FG discussions will be conducted at locations where the participants are based, for example, community centres, libraries, schools, village halls, food banks, religious groups and workplaces, as advised by members of our LEAG. Participants’ travel expenses to take part in the study will be reimbursed. FGs will act as a social space where participants share, acquire and contest knowledge to coproduce a situated view on unmet mental health needs in the population.^50^ Using topic guides, we will explore whether the preidentified priorities meet participants’ needs and identify any remaining gaps, for example, the impact of rural/coastal living or minoritised identities on mental health; hence, developing our understanding of the mechanisms through which our research programme strategy might lead to different mental health outcomes for different groups in our populations (see online supplemental material 2). Participants will be asked to list three mental health research priorities not included in the predefined priority list to capture any unanswered research priorities from their perspectives. They will be asked to rank their three mental health research priorities on a 3-point Likert Scale (0=low priority; 1=moderate priority; 2=high priority). Participants will also be asked to indicate their willingness to participate in the research priority-setting workshops. Discussions will be audio-recorded and transcribed. Participants will be offered a £25 gift voucher each to thank them for their time.

#### Sample size

Sample sizes are indicative and in keeping with previous studies.^48^ We will hold 10 FG discussions in total, that is, two per participant group with lived experience consisting of a minimum of four people per group, that is, (1) adults with lived experience, (2) family carers, (3) young people (16–25 years), (4) older people and (5) marginalised communities. An estimated sample of 40 participants will be recruited.

### Phase 3: collation—data analysis (study objective 3)

*Aim:* This phase aims to process the information from the survey and FG discussions. A team approach will be taken to analyse the data involving researchers, members of the LEAG and SG members. Training, support and mentorship in data analysis/interpretation will be provided to people with lived experience.

#### Data analysis

We will use a triangulation approach to integrate the data from the survey and FG discussion. First, we will analyse data from each data source separately using qualitative and quantitative methods as appropriate. As such, descriptive statistics will be used for data from the survey, and thematic analysis for data from FG discussions to identify common themes of high priorities. Data analysis will be conducted independently by two researchers. Second, a team of researchers, including our LEAG lead, will make sense of, compare and group the topics and questions from both data sources. Third, based on the triangulated results, the team will produce a shortlist of priorities/questions to be voted on and ranked during the 2-day prioritisation exercise, which will include representatives from our stakeholder and community groups. Triangulation is a recognised approach to achieving and maintaining consistency, validity and rigour in mixed methods research.^51^

### Phase 4: prioritisation (study objectives 3 and 4)

*Aim:* This phase aims to synthesise the information collected in Phases 2 and 3 and bring stakeholders together to agree on the top 10 research priorities for mental health to inform our MHRG research programme strategies.

#### Participants and sampling

Participants who have registered an interest in Phases 2 and 3 will be invited to participate. The JLA recommends the inclusion of 12–30 participants per research prioritisation workshop.^46^ We anticipate around 15 stakeholders per workshop will take part. Two participant groups will be included: (1) the service users and public group and (2) the professionals group. We will adapt our recruitment strategy to purposively target groups often under-represented in research.

#### Data collection

We are mindful that combining different groups with potentially conflicting ideas in a single workshop may undermine equal representation in the final consensus.^52^ To overcome this, we will run two in-person seminars over 2 days to rank and prioritise the research topics generated in Phase 3. The ranking exercise on day 1 will involve the service user and public group, and day 2 will involve the professionals group. Prior to each workshop, participants will be sent the shortlist of priorities identified in the previous phases. At each seminar, group discussion will focus on the relevance of the known mental health research priorities to Norfolk and Suffolk and the newly identified priorities. Two researchers will facilitate the workshops for consistency, and we will use a Nominal Group Technique, as per the JLA methodology,^46^ to group the shortlist. Participants will first be split into two groups of eight participants maximum to give their views about which research priorities are most or least important. They will be asked to rank or vote on their preferences into high (two points), medium (one point) or low (0 points) priority categories. The ranked total from each group will be summed, and the priorities with the highest score (ie, most favoured) total ranking will be selected as the top priorities. Participants will be returned to the large group to discuss the ranking and arrive at the finalist list of up to 10 mental health research priorities^17 46^ in Norfolk and Suffolk.

To ensure that lived experience and public view remain central to the priority-setting exercise, the professional group will receive the ranking scores and feedback from the lived experience panel. This adaptation has been used in previous studies, where evidence shows that providing feedback on patient scores to healthcare professionals results in an expanded set of consensus items that better reflect the priorities of patients.^52 53^ To minimise the possibility of members of the public workshop feeling pressured to tailor their answers/ranking, the public panel will not receive feedback on professional scores.^52^ Discussions will be audio-recorded and transcribed. Participants will be offered a £25 gift voucher each to thank them for their time. Travel expenses will also be covered.

### Phase 5: future research development and capacity building (study objectives 4 and 5)

*Aim:* This final stage aims to develop our research programme strategy, including capacity building and formally bringing our team together by setting out the structure of the Norfolk–Suffolk MHRG.

#### Procedure

**Figure 1:**
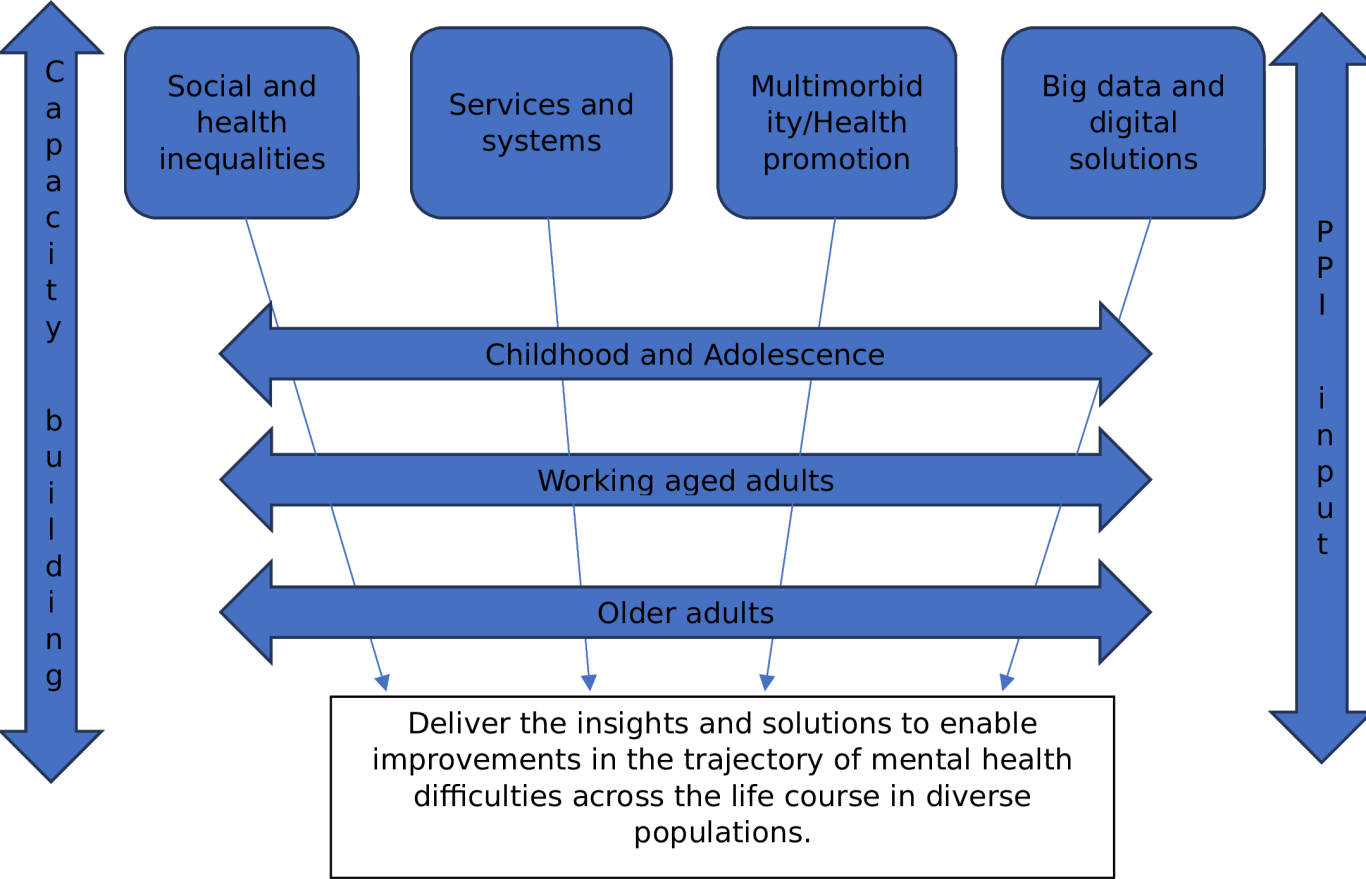
Flowchart of themes to be developed for the NIHR Norfolk–Suffolk MHRG. MHRG, Mental Health Research Group; NIHR, National Institute of Health and Care Research; PPI, patient and public involvement.

The research priority-setting in this development work will give us the knowledge required to develop a 5-year research programme that addresses the trajectory of mental health difficulties across the life course in Norfolk and Suffolk. Based on JLA priorities, we anticipate that the broad research themes emerging in Phases 1–4 that need to be included in our NIHR MHRG programmes of research could consist of the workstreams/themes shown in the road map in figure 1. The activities will be coproduced and codelivered with patient and public involvement input throughout.

## Patient and public involvement (PPI)

Our ultimate goal is to give people living with mental health difficulties, their families, health and social care professionals (including statutory and third sector providers), researchers and policymakers a voice in deciding the most important questions to be answered by future research. We have worked with people with lived experience, communities and partner organisations from Norfolk and Suffolk to design this study. The chief investigator (SO) met people with lived experience and several individuals from a range of local organisations (including charities supporting young people, women and agricultural communities) to gain their perspectives on the project idea. The members of this PPI group supported the importance and timeliness of the study. Those with lived experience of mental health difficulties shared their experiences of a lack of timely access to care, insufficient duration of treatment and a lack of opportunities in coastal areas. There was a consensus that separate prioritisation workshops be held with professional and user/public stakeholders to reduce the risk of service users feeling pressured to agree with the professionals. We have incorporated all the stakeholders’ suggestions and comments in the protocol.

We have convened an SG and a LEAG, with whom we will conduct the project over 12 months. To date, members of our LEAG have contributed to writing the plain English summary and designing the survey questionnaires and FG discussion/interview topic guide. The LEAG will be supported and led by a co-investigator who also brings a lived experience, and the group will be involved in all study aspects, including recruitment, interpretation of findings and dissemination. Crucially, the LEAG will be involved in developing our large research programme to ensure that the perspectives of experts by experience are captured. We will also explore and develop structures for peer researcher development. LEAG members will be reimbursed according to NIHR recommended rates.

## Ethical considerations and data management

Participation in the study is voluntary. All participants will be given written and, where possible, verbal information about the project to allow them to make informed decisions about their participation. The online survey will ask participants to confirm their participation via an embedded consent statement. FGs and prioritisation workshops will take place in person. Participants will be asked to sign a consent form; researchers will remind them that they can withdraw anytime. We will make participants aware that once data are anonymised, withdrawal/removal may not be possible. All efforts will be made to ensure confidentiality. However, we will remind participants not to share sensitive information because confidentiality cannot be guaranteed, given the nature of FG discussions and workshops. FG and prioritisation workshop participants will be offered a £25 gift voucher each as reimbursement for their time in taking part. We will also cover the travel expenses made by participants to take part in the study. Participants in the online survey will have a chance to win one of twenty £25 gift vouchers as reimbursement for their time for completing the questionnaire.

The General Data Protection Regulation and the UEA Research Data Management Policy (version 1.7) (UEA, 2019) will be fully adhered to. Data from the online survey, FG discussions and workshops will be stored on a password-protected UEA server. Following study completion, the data will be held for 10 years at the UEA, after which it will be destroyed.

Further, we have developed a safeguarding procedure for participants who might be at risk. For example, suppose a participant becomes distressed during the group discussion or interview. In that case, the researcher will stop the interview, and immediate support will be provided along with signposting to sources of additional support (eg, general practitioner, care coordinator and appropriate voluntary organisation). Additionally, if any information provided concerns the research team that a participant is at risk or if they provide information that others might be at risk. A decision on how to proceed in such circumstances will be made in consultation with the study chief investigator, who is an experienced mental health nurse, and, if possible, agreement will be reached with the participant on the steps to be taken.

This study was approved by the UEA’s Faculty of Medicine and Health Research Ethics Committee (reference: ETH2324-2542), Norwich, UK. The ethical conduct of the study is monitored throughout by the UEA.

## Dissemination and anticipated impact

We will leverage our strong network of stakeholders and partners involved in mental health services, such as people with lived experience, academics, clinicians, policymakers, the third sector and government and non-government organisations. Knowledge mobilisation and dissemination strategies will be tailored to specific stakeholders, working closely with our LEAG and SG to produce newsletters, websites, short summaries, publications and conferences. The LEAG members and SG contributions will be recognised in all dissemination, including coauthorship of article publications. The CSMH will support the development of a collaborative research programme strategy for the MHRG Award, providing mentorship, sharing approaches to and experiences of building partnerships and supporting capacity-building activities. The potential impact of this project and (if funded) the subsequent larger programme of research will include improving health and reducing mental health burdens (eg, narrowing treatment gaps, reducing hospitalisation/emergency admissions, improving access to care, increasing social connection) and economic impacts, such as saving the NHS money.

In summary, this study will use a mixed-methods approach to consult and collaborate with people with lived experience with mental health difficulties, mental health experts, policymakers and members of the public to identify priorities and capacity gaps for mental health research in Norfolk and Suffolk. Its strengths are in the multidisciplinary and interdisciplinary partnership between, and the involvement from inception of, key local and regional stakeholders and a strong collaboration with the CSMH King’s College London. Our systematic and transparent approach to the research priority setting aligns with other evidence-based priority research activities and a strong commitment to dissemination and future research. The priorities identified from the work will lay the foundations for larger programmes of research intended to address unmet needs and improve mental health outcomes.

## supplementary material

10.1136/bmjopen-2024-093980 online supplemental file 1

10.1136/bmjopen-2024-093980 online supplemental file 2
