# Supplementary material for: Changing the trajectories of mental health difficulties in Norfolk and Suffolk: a research-priority-setting project with patients, the public, clinicians, policymakers and other stakeholders—study protocol
Source: BMJ Open. 2025 Jan 4;15(1):e093980. doi: 10.1136/bmjopen-2024-093980 (PMC11749443; doi:10.1136/bmjopen-2024-093980)
Supplement: online supplemental file 1 [file bmjopen-15-1-s001.pdf]

MS form banner: ***Help us find the top 10 research priorities for mental health in Norfolk & Suffolk***

About this research project

Project title: Changing the trajectories of mental health difficulties in Norfolk and Suffolk: a research-priority-setting project with patients, the public, clinicians, and stakeholders.

We want to find out the most important gaps in knowledge about mental health difficulties and hear what matters to you in providing the best mental healthcare in Norfolk and Suffolk, particularly in rural, coastal, and migrant communities.

This survey is the first part of a larger project. Our main goal is to develop research that can address mental health problems at every stage of life. We want to look at things we can do to prevent ill health, promote positive mental health and well-being, and reduce health inequalities in Norfolk and Suffolk. This survey aims to identify the key mental health research priorities for our unique communities. We will gather the perspectives of service users, the public, communities interested in mental health and mental healthcare.

At national and international levels, mental health research priorities exist, e.g., those of the World Health Organisation, the James Lind Alliance, and other research groups. We want to see whether these existing research questions are relevant to people in our unique rural/coastal areas. The survey will ask you to say which of the suggested research questions you think are the most important by ranking them on a scale. We also invite you to write your ideas and suggestions for priority areas not included in the pre-defined questions.

The final Top 10 priorities will be decided in a 2-day workshop in late Autumn 2024. If you would like to take part in the priority-setting workshop, we will ask you to give your contact details at the end of the survey. We will address the top 10 priorities in a larger research programme for the next five years, for which we hope to receive funding in 2025/26.

Who is conducting the research?

Researchers at the University of East Anglia are running the project in collaboration with the Centre for Society and Mental Health at King's College London. We also are working with a Steering Group consisting of people who have experienced mental health issues, healthcare professionals, and researchers. This research is funded by the National Institute for Health and Care Research (NIHR).

Why have I been invited?

We are inviting you to take part if you are aged 16 years or over and:

- someone who has experienced or is experiencing mental health difficulties.
- a partner, family member or friend of someone who has experienced or is experiencing mental health difficulties.
- a mental health professional, mental healthcare student or support worker.
- a representative of an organisation or charity which supports people with their mental health.
- a policymaker, researcher, educator, employer
- a member of the public who is interested in mental health.

What is involved?

The survey asks you to rate how important you think different areas of mental health research are. We encourage you to answer as many questions as you can. However, when filling out the questionnaire, if you do not feel comfortable answering a particular question for any reason, you are welcome to skip the question and move on. The survey should take 20 to 30 minutes to complete. Please try to complete this in one single session.

Will there be any risks or other implications of taking part in this project?

We are not aware of any risks or health implications to you by taking part in this research. No identification data (name, address etc.) will be collected about you and all data collection will be held confidentially and securely. Should answering the questions about your mental health upset you or cause you distress, we will provide information about sources of support at the end of the survey. Should you have any concerns about your own mental health, we suggest you talk to your GP or a specialised mental health support service. We have listed some services below:

- The Samaritans (a safe space to talk) are available on 116 123. You can also download their Self-Help app onto your phone or email jo@samaritans.org, and they will respond within 24 hours.

- UEA student services: Wellbeing - https://my.uea.ac.uk/divisions/student-services/wellbeing.
- Crisis support - https://my.uea.ac.uk/divisions/student-services/wellbeing/crisis-support.
- NHS Mental Health support - https://my.uea.ac.uk/divisions/student-services/wellbeing/mental-health/nhs-mental-health-support.

What are the benefits of taking part?

While there are no immediate benefits for those taking part in the project, this work will benefit the development of large-scale research which will address the identified priorities. We hope that this work will reduce mental health problems, improve lives, and promote well-being in our communities.

Will my involvement be confidential?

Yes. All information collected will be anonymous and stored in compliance with the 2018 Data Protection Act and the 2018 General Data Protection Regulation. It will be stored on a secure UEA computer and will only be looked at by the research team. The findings of the research may be used in academic publications, conference presentations, reports for external organisations and on websites. Research data will be stored for 10 years and then destroyed.

Will I be paid to take part?

There is no payment for taking part in the survey. However, there is an optional prize draw where you could win one of twenty £25 Amazon gift vouchers. To enter the prize draw, you can use the weblink at the end of the questionnaire to enter your email address. This is so we can securely store your email address separately from your survey responses and protect your anonymity.

Who has reviewed the research?

The University of East Anglia (UEA) Faculty of Medicine and Health Sciences (FMH) Ethics Committee has been asked to give ethical approval for this study [Reference: ETH2324-2542].

This survey closes at midnight on Sunday 24 November 2024.

Consent

Please confirm that you have read and understood the information above, and you are happy to proceed (mandatory).

- Yes
- No

Information about you

Q1 Which of these options best describes you in terms of your participation in this research? Please select more than one option, if applicable

A. Someone with a lived experience of mental health difficulties
B. Partner, family member or friend of someone with a lived experience of mental health difficulties.
C. Mental health professional, healthcare student or support worker.
D. Representative of an organisation or charity which supports people with their mental health.
E. Policymaker, researcher, educator, employer
F. Member of the public who is interested in mental health.

Q2 If you answered A to question 1, please complete questions 2a to 2d:

Q2a, Do you have a mental health diagnosis?

- Yes
- No

Q2b if yes to 2a, did you receive the diagnosis from an NHS or private healthcare professional e.g., GP, clinical psychologist, or psychiatrist?

- Yes
- No
- Other (please state)

Q2c How long have you had the diagnosis?

(Please write in months/years)

Q2d Are you currently receiving treatment (e.g., taking medication of your mental health or seeing a healthcare professional)?

- Yes
- No

Q3 Are you currently employed?

- Yes
- No

Q3b What is your current job title/role? Please write:

Q4 What is your age group? (Please select the appropriate button group)

- 16-19
- 20-29
- 30-39
- 40-49
- 50-59
- 60-69
- 70-79
- 80-89
- 90+

Q5 What is the sex assigned to you at birth?

- Male
- Female

Q6 Which one of these options best describes your gender?

- Woman
- Man
- Non-binary
- Transgender
- Other (please specify)
- Prefer not to say.

Q7 Which of the following best describes your ethnicity?

1. English/ Welsh/ Scottish/ Northern Irish/ British
2. Irish
3. Gipsy, Traveller or Irish Traveller
4. Any other white background
5. White and Black Caribbean
6. White and Black African
7. White and Asian
8. Any other mixed/ multiple ethnic background
9. Indian
10. Pakistani
11. Bangladeshi
12. Chinese
13. Any other Asian background
14. Caribbean

15. African
16. Any other black/ African/ Caribbean background
17. Arab
18. Any other ethnic background
19. Prefer not to say.

Q8 What is your country of birth? Please write:

Q9 Do you live or work in East Anglia?

- Yes
- No

Q9a (If answered Yes to Q9) Where do you currently live?

- Norfolk
- Suffolk
- Other (please state)

Q10 Have you moved to the East Anglia region in the last 10 years?

- Yes
- No

Q10a If you have moved to the area, where did you move from:

- within the UK
- outside the UK
- Prefer not to say.

Q10b If you have moved from outside the UK, which region did you move from?

- EU country
- Other European country
- Commonwealth country
- Middle East
- Africa
- Asia
- North America
- South America
- Prefer not to say

Q11 Which one of these options best describes the area you currently live?

- Urban area (city or large town of over 10,000 people)
- Rural town and fringe (small town of less than 10,000 people, or in countryside)
- Coastal area (seaside, near the shoreline of sea or ocean)
- Hamlet/village (small settlement of less than 1,000)

Q12 Which of these—if any—is your highest educational or professional qualification? Please choose the highest option on the list that applies to you. If you are still studying, choose the highest qualification you have completed so far.

1. I have no formal qualifications (and I am not still studying)
2. GCSE/ O' Level/ CSE/ National Qualifications/ Standard Grades
3. Level 1-3 vocational qualification or intermediate – advanced apprenticeship
4. A' level, Scottish Higher, Welsh Baccalaureate, International Baccalaureate or equivalent)
5. Diplomas in higher education (HNC/ HND/ BTEC Higher or equivalent)
6. Level 4-5 vocational qualification or higher apprenticeship
7. University first degree (BA/ BSc/ BEd/ PGCE or equivalent)
8. University higher degree (e.g. Masters, PhD or equivalent)
9. Still studying
10. I prefer not to say

Research priority questions.

Thank you very much for your answers so far.

We will now present research priority statements in seven sections addressing the key domains below. Within each section, there are 10 priority statements.

A. Children and Young people's mental health (up to 25 years old)
B. The link between physical and mental health
C. The impact of rural and coastal living on mental health
D. Access to mental healthcare
E. Migration and mental health
F. The impact of social and health inequalities on mental health
G. Mental health promotion and prevention

Note: we use the term mental health difficulties to capture any diagnosable mental health condition, e.g., depression, schizophrenia, psychosis, personality disorders, addiction, suicide, anxiety, eating disorders, learning disabilities, bipolar disorder, attention deficit hyperactivity disorder (ADHD), and autistic spectrum disorders.

Section A: Children and Young people's mental health.

Q1. In this section, we present research priority statements for children and young people's mental health. Please rank how important each statement is to you as follows: (0= low priority; 1 = moderate priority; 2= high priority)

Research priority statements	0= low priority	1= moderate priority	2= high priority
Involve young people in making decisions about their mental health treatment			
Increase availability of effective culturally appropriate services in children and young people's mental health, particularly for ethnic minority groups			
Training teachers and other staff in schools and colleges to detect early signs of mental health difficulties in children and young people			
Child and Adolescent Mental Health Services (CAMHS), education providers, and health and social care departments work together more effectively to improve the mental health outcomes of children and young people			
Supporting young people on Child and Adolescent Mental Health Services (CAMHS) waiting lists to prevent further deterioration of their mental health			
Early intervention strategies for supporting children and young people in improving their mental resilience			
Impact of parental attitudes to mental health on the treatment outcomes of children and young people with mental health difficulties (both positively and negatively)			
Understanding the impact of immigration policy and the immigration process on the mental health of young people who are migrants			
Screening young people for the early identification of mental health difficulties			

Q2. Within which setting would screening of young people for mental health difficulties be most effective? Please rank each of the following:

- Schools/colleges
- GP
- Voluntary sector (charities, community centres)
- Social Care
- Mental health Trust
- General hospitals

Section B: The link between physical and mental health

Q1. This section presents research priority statements on the link between mental and physical health. Please rank how important each statement is to you as follows: (0= low priority; 1 = moderate priority; 2= high priority)

Research priority statements	0= low priority	1= moderate priority	2= high priority
Treating mental and physical health together			
Primary care services supporting people with antipsychotic-induced weight gain			
Managing the side effects of psychotropic medications (including weight gain, problems with thinking and memory, and emotional numbness)			
Identifying people with mental health difficulties at high risk of type 2 diabetes and preventing the condition from developing			
Identifying the most effective and acceptable strategies for preventing multiple physical and mental health conditions in later life			
Establishing a greater understanding of the link between mental health conditions and cardiovascular diseases.			
Improving management and care for physical health problems in mental health inpatient care			
Identifying interventions that are most effective in improving sleep in people with mental health difficulties			
Identifying people at high risk of rapid weight gain during the first episode of psychosis			
Improving physical health checks in people living with mental health difficulties			

Section C: Access to mental healthcare

Q1. We now present statements around access to mental health care. Please rank how important each statement is to you as follows: (0= low priority; 1 = moderate priority; 2= high priority)

Research priority statements	0= low priority	1= moderate priority	2= high priority
Better awareness of where to seek help for mental health difficulties.			
Improving access to mental health care for rural and coastal communities			
Improving access to mental health care through digital interventions			
Improving access to mental health care for all age groups			
Understanding which models of care increase access and support decision-making for people with mental health difficulties			
Improving access to psychological therapy-style services for children and adolescents			
Increasing access to psychological/emotional support for family carers/informal carers of people with mental health difficulties			
Increasing access to care for people who don't speak or read English			
Integrating the work of mental health charities with NHS services to improve access to care			
Addressing the barriers to accessing care and treatment for mental health difficulties in rural, remote and coastal areas			

Thank you for your responses so far. You are approximately halfway through the survey.

Section D: Impacts of rural and coastal living

Q1. We will now ask you to rank statements around the impact of rural and coastal living on mental health in the order of importance to you as follows: (0= low priority; 1 = moderate priority; 2= high priority)

Research priority statements	0= low priority	1= moderate priority	2= high priority
More collaboration between health, social care, and voluntary sectors to deliver mental health care to people in rural areas			
Understanding the impacts of social isolation on physical and mental health			
Overcoming the stigma of mental illness in rural areas			
Understanding the benefits of rural/coastal living for mental health			
Understanding the impacts of climate emergency on mental health in coastal communities			
Increase social and economic opportunities to enhance the mental health and wellbeing for young people and adults living in rural and coastal communities.			
Increase healthcare professionals' training in and awareness of the mental health needs of those living in rural and coastal communities			

Q2. Understanding the impact of rural/coastal living on (Please rank how important each phrase below is to you as follows: (0= low priority; 1 = moderate priority; 2= high priority)):<br>• waiting times,<br>• preferred appointment times<br>• recovery			

Q3. At what point in the care pathway are digital interventions most safe and effective in rural areas? (Please consider times when people need urgent care, ongoing treatment and how to maintain good

mental health) Please rank how important each phrase is to you as follows: (0= low priority; 1 = moderate priority; 2= high priority)):

- Crisis intervention
- Prevention
- Engagement
- Treatment
- Maintenance and recovery

Q4. If there was a single service that provides mental health support in rural/coastal areas, where should it be physically located? Please rank each:

- Library
- GP surgery
- Local charity
- Community Centre
- Village hall
- Church hall
- Mental health team base
- Pharmacist/Chemist

Q4b. Are there any other physical locations where mental health support should be provided in rural/coastal areas? Please state:

Section E: Migration and mental health

Q1. In this section we present statements on the impact of migration on mental health. Please rank each statement in the order of importance to you as follows: (0= low priority; 1 = moderate priority; 2= high priority)

Research priority statements	0= low priority	1= moderate priority	2= high priority
Experiences of migration among older people living in rural and coastal areas			
Understanding the impact of immigration policy and the immigration process on the mental health of migrant groups			
Experiences of migrants in the health and social care workforce			
Understanding which factors affect the mental health and wellbeing of migrants after moving to the UK			
Supporting migrant health and social care workers in their roles to improve their mental health			
Develop community-level interventions to improve the mental health of migrant groups			
Developing more evidence on the impact of immigration policy on migrant's right to work or access to social housing and how this affects mental health and wellbeing			
Learning from previous cohorts of international migrants on how mental health services can better support migrants' mental health			
Understanding the influence of health literacy among migrants on access to the UK health system			
Understanding the positive and negative impacts of migration within the UK on mental health			

Section F: Social and health inequalities

Q1. In this section we present statements relating to social and health inequalities on mental health. Please rank each statement in the order of importance to you as follows: (0= low priority; 1 = moderate priority; 2= high priority)

Research priority statements	0= low priority	1= moderate priority	2= high priority
Improving access to mental health care for ethnic minority groups			
Provision of talking therapy in languages other than English			
Increase health and social care professionals training in and awareness of mental health needs in LGBTQ+ communities			
Improving perinatal mental health care for ethnic minority women			
Develop culturally sensitive care for racial and ethnic minoritized groups			
Understanding how effective community-based interventions are in improving mental health in deprived areas			
Interventions for reducing involuntary hospital admissions among black and ethnic minoritised groups			
Identify mental health policy/interventions that could target key stages of life (over the life course) that could prevent poor mental health outcomes			
Develop public health interventions to ensure no one is 'left behind'			

Q2. Please rank the following population groups in order of priority for improving prevention efforts for suicide and self-harm.

- Women of South Asian origin
- LGBTQ+ people
- Farmers and agricultural communities
- Other jobs with higher risk of suicide

- Young people
- Older people
- Men
- People with disabilities
- Ethnic minority groups
- Migrants

Section G: Mental health promotion and prevention

Q1. We will now ask you about mental health promotion and prevention. Please rank each statement in the order of importance to you as follows: (0= low priority; 1 = moderate priority; 2= high priority)

Research priority statements	0= low priority	1= moderate priority	2= high priority
Developing risk prediction tools for mental health difficulties			
Increase awareness of risks around pregnancy and childbirth in women people with psychosis, depression and bipolar disorder.			
Support to find and retain employment among adults with mental health difficulties			
Implementation of peer support systems across mental health services			
Support for carers of older people with multiple conditions to maintain their psychological well-being			
Understanding how mental health difficulties impact employment			
Supporting parents or family members in identifying early signs of mental health difficulties in children and young people.			
Empowering patients, relatives, and carers be to have a greater say about their choices regarding mental health and mental healthcare			

Q2. Please rank the following types of de-identified data (e.g., health, education, local authority records) based on their potential to help improve mental health outcomes in rural and coastal areas:

- Identifying risk factors of mental illness
- Examining mental health outcomes
- Evaluating services against agreed health outcomes and policies

Q3. Please rank the following population groups in order of priority for initiating early intervention to help prevent mental health problems.

- LGBTQ+
- ethnic minorities
- older people
- people with disabilities
- migrants

Q4.. Please write up to three topics or questions not included in the list above that you would like us to consider in future research. (3 free text lines)

Thank and close

That is the end of the survey now; thank you very much for your time.

Further research opportunities and Prize draw

If you wish to be entered into the £25 Amazon voucher prize draw or register your interest in further opportunities to be involved in the research, please use this link to enter your details - https://forms.office.com/e/v9LZQHMPSS This takes you to a new page so we can keep your survey responses separate from your email address to protect your anonymity.

Debrief form:

Thank you for taking part in this study. The aim of this research is to define 10 top mental health priorities for Norfolk and Suffolk populations.

If you feel concerned about your mental health, please speak with your GP. If you are feeling distressed as a result of taking part in the survey and feel you are in crisis, please contact the Samaritans (a safe space to talk) at any time on 116 123, call 999 or go to A&E. You can email jo@samaritans.org for a response within 24 hours or download their Self-Help app onto your mobile phone.

If you have any questions about the study, please contact Sol Morrissey the study co-ordinator by email at mental.health@uea.ac.uk

If you have any questions that cannot be answered by Sol, or if you have any concerns about how the research is being done, please contact Prof. Lee Shepstone at UEA who is independent of this research. They can be contacted by email L.Shepstone@uea.ac.uk or phone 01603 592100.
