# Supplementary material for: Changing the trajectories of mental health difficulties in Norfolk and Suffolk: a research-priority-setting project with patients, the public, clinicians, policymakers and other stakeholders—study protocol
Source: BMJ Open. 2025 Jan 4;15(1):e093980. doi: 10.1136/bmjopen-2024-093980 (PMC11749443; doi:10.1136/bmjopen-2024-093980)
Supplement: online supplemental file 2 [file bmjopen-15-1-s002.pdf]

Qualitative Focus Group/ Interview Topic Guide

Changing the trajectories of mental health difficulties in Norfolk and Suffolk: a research-priority-setting project with patients, the public, clinicians, policymakers, and stakeholders

YOUNG PERSON/PEOPLE

A. Introduction

- Welcome
- Explanations, answer questions, consent
- Remind of confidentiality and anonymity
- Invite to complete the online survey before (or after, if preferred) the focus group
- Complete demographic data form (i.e., age, gender, residential context (rural/coastal), occupation, etc.)

B. Experience of mental health difficulty and help-seeking

Scene setting:

- What does mental health mean to you?

Prompt: draw examples from everyday life, feelings, emotions, well-being, hobbies, etc.

- In your opinion, how would you describe a mental health difficulty or problem?

Can you recall when you first felt you needed to seek help for a mental health difficulty? Whom did you see (e.g., GP, hospital, psychiatrist, family/friend, charity organisation)?

Prompt:

- ➢ *Tell me (us) about that experience. Was it a good or bad experience?*
- ➢ *In your opinion, how can help-seeking and access to mental health support be improved?*

C. Impact of rural/coastal living

- What do you think is the biggest mental health gap in Norfolk and Suffolk today?

Prompt:

- ➢ *Why do you think these gaps exist in our communities?*
- ➢ *Do you think people with mental health difficulties experience different challenges depending on whether they live in urban, coastal,* or *rural areas?*
- ➢ *From your perspective, which group(s) of people are at high risk of poor mental or physical health in rural and coastal communities?*
- According to previous research, people living in rural areas (for example, a village or town) in the UK have less chance of accessing mental health services. Do you have any opinion about the possible reasons for this based on your own experiences?
- Research studies and government reports show that in some coastal areas, young people have limited job opportunities, which can affect their mental health. What are your views on this?

Prompt:

- ➢ *What other aspects of living circumstances (housing, relationships, physical environment, climate change, coastal erosion) do you think could affect your mental health? Tell me more about that.*

- What are your views on the stigma around mental health, especially in rural communities?

Prompt:

- ➢ *In your view, how can we reduce the stigma of mental health?*
- ➢ *Can you share examples of great anti-stigma campaigns that you've come across?*

Potential solutions

- What kind of things can we do to help prevent young people from experiencing mental health difficulties in the first place?
- How do you think mental health services can be improved in rural and coastal areas?
- If there was a single service that provided all the help you need, what would it look like?

Prompt:

- ➢ *Who could give this type of service? (GP, nurses, psychologists, doctors, other healthcare professionals, charity workers,*
- ➢ *Where would you feel okay getting this kind of help/service? (Charity organisations, healthcare services, schools/colleges, workplace)*
- ➢ *How would we know if it was working?*

D. Health inequalities

- Do you feel that the needs of people with protected characteristics (LGBTQ+, ethnic minority groups, older people, etc.) are generally met by healthcare services?

Prompt:

- ➢ *What do you think the gaps are in meeting the mental health needs of minority groups (LGBTQ+, ethnic minority groups, older people, etc.) and of different ages?*
- In your opinion, how can we better support people with protected characteristics in our communities to ensure their mental health does not deteriorate?

  Prompt:
- ➢ *Can you give me some examples of helpful things that you have come across?*

For migrants

- In recent years, the number of people moving (from abroad and within the UK) to Norfolk and Suffolk has gone up. Research shows that migration can be a stressful experience which can affect mental health. Do you have any opinion about the possible impact of migration on mental health based on your own experiences?

Prompt:

- ➢ *What are your views on loneliness and social isolation as a result of migration?*
- ➢ *Are there ways we (mental health providers) can better support migrants so that their mental health does not deteriorate? Tell us about your views on this.*

E. Conclusion

- Is there anything we didn't talk about and which you think we should discuss?
- Of all the topics we have covered today, which do you think were most relevant to you?
- In your own words, tell us about the three most important areas of mental health in your community that could be improved through research.

Words of thanks and appreciation

Debrief: We've talked about a lot of things. How are you feeling after talking about all of this?

Prioritisation workshop

We will hold a two-day research prioritisation workshop in late Autumn 2024 to vote on and finalise the top 10 mental health research priorities. Would you like to take part in the workshop? If so, please write down your name and email address so we can contact you

FAMILY CARER(S)/FRIEND(S)

A. Introduction

- Welcome
- Explanations, answer questions, consent
- Remind of confidentiality and anonymity
- Invite to complete the online survey before (or after, if preferred) the focus group.
- Complete demographic data form (i.e., age, gender, residential context (rural/coastal), occupation, etc.)

B. Experience of mental health difficulty and help-seeking

- Scene setting
- What does mental health mean to you?

Prompt with everyday examples if participant thinks of high-end disorder.

- In your opinion, how would you describe a mental health difficulty or problem?
- Can you recall when your child/relative/friend first needed to seek help for a mental health difficulty? Whom did they see (e.g., GP, hospital, psychiatrist, charity organisation)?

Prompt:

- ➢ *Did you help/encourage them to seek help from mental health services or elsewhere? Tell me (us) about that experience, was it good or bad?*

- In your opinion, how can help-seeking and access to mental health support be improved?

C. Impact of rural/coastal living and health inequalities

- What do you think is the biggest health problem facing our rural and coastal communities today?

Prompt:

- ➢ *Why do you think these gaps exist in our communities?*
- ➢ *From your perspective, which group(s) of people are at high risk of poor mental or physical health in rural and coastal communities?*

- According to previous research, people living in rural areas (for example, a village or town) in the UK have less chance of accessing mental health services. Do you have any opinion about the possible reasons for this based on your own experiences?
- Research studies and government reports show that in some coastal areas, young people have limited job opportunities (high unemployment), which can affect their mental health. What are your views on this?

Prompt:

- ➢ *What other aspects of living circumstances (housing, relationships, physical environment, climate change, coastal erosion) do you think could affect your mental health? Tell me more about that.*

- What kind of things can we do to help prevent people from experiencing mental health difficulties in the first place?
- What kind of support would you want if your child, friend or relative was showing signs of a mental health crisis?
- How do you think mental health services can be improved in rural and coastal areas?
- If there was a single service that provided all the help you need, what would it look like?

Prompt:

- *Who could give this type of service? (GP, nurses, psychologists, doctors, other healthcare professionals, charity workers,*
- *Where would you feel okay going to get this kind of help? (Charity organisations, healthcare services, schools/colleges, workplace)*
- *How would we know if it was working?*

- What are your views on the stigma around mental health, especially in rural communities? In your opinion, how can we overcome this?

D. Health inequalities

- Do you feel that people have equitable access to healthcare?
- Do you feel that the needs of people with protected characteristics (LGBTQ+, ethnic minority groups, older people, etc.) are generally met by healthcare services?

Prompt:

- *What do you think the gaps are in meeting the mental health needs of minority groups (LGBTQ+, ethnic minority groups, older people, etc.) and of different ages?*

- In your opinion, how can we better support people with protected characteristics in our communities to ensure their mental health does not deteriorate?
  Prompt: Can you give me some examples of helpful things that you have come across?

For migrants

- In recent years, the number of people moving (from abroad and within the UK) to Norfolk and Suffolk has gone up. Research shows that migration can be a stressful experience which can affect mental health. Do you have any opinion about the possible impact of migration on mental health based on your own experiences?

Prompt:

- *What are your views on loneliness and social isolation as a result of migration?*
- *Are there ways we can better support migrants so that their mental health does not deteriorate? Tell us about your views on this.*

E. Conclusion

- Is there anything we didn't talk about that you think we should discuss?
- Of all the topics we have covered today, which were most relevant to you?

In your own words, tell us about the three most important areas of mental health in your community that could be improved through research.

Words of thanks and appreciation
Debrief: We've talked about a lot of things. How are you feeling after talking about all of this?

Prioritisation workshop

We will hold a two-day research prioritisation workshop in October 2024 to vote on and finalise the top 10 mental health research priorities. Would you like to take part in the workshop? If so, please write down your name and email address so we can contact you.

ADULT/OLDER ADULTS

A. Introduction

- Welcome
- Explanations, answer questions, consent
- Remind of confidentiality and anonymity
- Invite to complete the online survey before (or after, if preferred) the focus group.
- Complete demographic data form (i.e., age, gender, residential context (rural/coastal), occupation, etc.)

B. experience of mental health difficulty and help-seeking

- Scene setting
- What does mental health mean to you?

Prompt with everyday examples if participant thinks of high-end disorder.

- In your opinion, how would you describe a mental health difficulty or problem?
- Can you recall when you first felt you needed to seek help for a mental health difficulty? Whom did you see (e.g., GP, hospital, psychiatrist, family/friend, charity organisation)?

Prompt:

- ➢ *Tell us about that experience. Was it good or bad?*

- In your opinion, how can help-seeking and access to mental health support be improved?

C. Impact of rural/coastal living

- What do you think is the biggest health problem facing our rural and coastal communities today?
- According to previous research, people living in rural areas (for example, a village or town) in the UK have less chance of accessing mental health services. Do you have any opinion about the possible reasons for this based on your own experiences?
- We also know from research that older people living in rural and coastal areas are vulnerable to poor health for a range of reasons, including social isolation, poor access to healthcare, lack of transport, etc. In your opinion, what are the challenges older people living in coastal areas face in maintaining good mental and physical health?

Prompt:

- ➢ *What other aspects of living circumstances (housing, relationships, physical environment, climate change, coastal erosion) do you think could affect your mental health? Tell me more about that.*

- What kind of support would you want if you were experiencing a mental health crisis?
- From your perspective, how can mental health services be improved in rural and coastal areas?
- If there was a single service that provided all the help you need, what would it look like?

Prompt:

- ➢ *Who could give this type of service? (GP, nurses, psychologists, doctors, other healthcare professionals, charity workers,*
- ➢ *Where would you feel okay going to get this kind of help? (Charity organisations, healthcare services, schools/colleges, workplace)*
- ➢ *How would we know if it was working?*

D. Health inequalities

- Do you feel that the needs of people with protected characteristics (LGBTQ+, ethnic minority groups, older people, etc.) are generally met by healthcare services?

Prompt:

- ➢ *What do you think the gaps are in meeting the needs of minority groups (LGBTQ+, ethnic minority groups, older people, etc.) and people of different ages?*

- In your opinion, how can we better support people with protected characteristics in our communities to ensure their mental health does not deteriorate?

Prompt:

- ➢ *Can you give me some examples of helpful things that you have come across?*

For migrants

- Research shows that migration can be a stressful experience which can affect mental health. In recent years, the number of people moving (from abroad and within the UK) to Norfolk and Suffolk has gone up. Do you have any opinion about the possible impact of migration on mental health based on your own experiences?

Prompt:

- ➢ *What are your views on loneliness and social isolation due to migration?*
- ➢ *Are there ways we can better support migrants so that their mental health does not deteriorate? Tell us about your views on this.*

E. Conclusion

- Is there anything we didn't talk about that we should discuss?
- Of all the topics we have covered today, which were most relevant to you?
- In your own words, tell us about the three most important areas of mental health in your community that could be improved through research.

Words of thanks and appreciation
Debrief: We've talked about a lot of things. How are you feeling after talking about all of this?

Prioritisation workshop
We will hold a two-day research prioritisation workshop in late Autumn 2024 to vote on and finalise the top 10 mental health research priorities. Would you like to take part in the workshop? If so, please write down your name and email address so we can contact you.
