# Supplementary material for: Changing the trajectories of mental health difficulties in Norfolk and Suffolk: a research-priority-setting project with patients, the public, clinicians, policymakers and other stakeholders—study protocol
Source: BMJ Open. 2025 Jan 4;15(1):e093980. doi: 10.1136/bmjopen-2024-093980 (PMC11749443; doi:10.1136/bmjopen-2024-093980)
Supplement: Supplementary file 3 [file bmjopen-2024-093980.reviewer_comments.pdf]

PEER REVIEW HISTORY

BMJ Open publishes all reviews undertaken for accepted manuscripts. Reviewers are asked to complete a checklist review form and are provided with free text boxes to elaborate on their assessment. These free text comments are reproduced below.

ARTICLE DETAILS

Title (Provisional)

Changing the trajectories of mental health difficulties in Norfolk and Suffolk: a research-priority-setting project with patients, the public, clinicians, policymakers, and other stakeholders- study protocol

Authors

Oduola, Sherifat; Morgan, Craig; Das-Munshi, Jayati; Broomfield, Niall; Parretti, Helen; Sanderson, Kristy; Notley, Caitlin; Zile, Amy; Morrissey, Sol; Khadjesari, Zarnie; Holmes, Joni

---

VERSION 1 - REVIEW

**Reviewer**	**1**
**Name**	**Cusworth Walker, S**
**Affiliation**	**University of Washington School of Medicine, Psychiatry and Behavioral Scieences**
**Date**	**03-Nov-2024**
**COI**	**None**

Thank you for the opportunity to review this study protocol regarding a participatory, research setting priority project with multisector, diverse constituents. The protocol was well-written and organized.

Introduction. The introduction provides well-justified arguments and citations regarding the value of focusing on UK coastal areas for mental health research and practice/policy intervention. The authors note the value of addressing disparities in research, but could also include more examples and citations of how community-engaged research leads to public impact. For example, the authors could reference the literature on Research Practice Partnerships, or community participatory research resulting in collaborative policy advocacy between researchers and community members. The intended or hypothesized link implicit in the researchers' laudable effort could be more explicit.

Methods. The methods were clearly described and included appropriate procedures for the researchers' aims, including multiple methods of capturing feedback from community members as part of initial prioritization. The researchers will want to take care that

participation in in-person ranking exercises attempts to include those who may not routinely volunteer for those types of activities.

Aims. The phases of the study proceed logically and thoughtfully. Again, the researchers might consider being more explicit in how research priorities are expected to achieve practical changes as this may influence the types of capacity needed for the ongoing collaboration.

Congratulations to the authors on an exciting venture.

**Reviewer**	**2**
**Name**	**Whitehorne-Smith, Patrice**
**Affiliation**	**Curtin University Faculty of Health Sciences**
**Date**	**04-Nov-2024**
**COI**	**None**

This is a well designed and detailed protocol. It appears to be a highly relevant study. The methodology and strategies for inclusion of all critical stakeholders creates the foundation for a highly impactful and actionable body of research. I have three minor suggestions:

1. The introduction could be strengthened by bringing in literature related to the social determinants of mental health. Although some of these factors are alluded to, clearly demonstrating the factors you have identified within that framework would further justify the need for the inclusion of diverse stakeholders in the study and the intersectionality implied in your approach.

2. The introduction highlights an influx of immigrants within Norfolk and Suffolk, however your methods does not include an specific language based exclusion criteria. Will fluency in English be a consideration for participation in the study?

3. Phase 2b-in person survey and focus group discussions- although it was stated that focus groups will be conducted where participants are based, it is unclear if the travel costs to the location for the purpose of the focus group will be covered in this phase. Travel expenses can be a major deterrent to the participation of marginalised or vulnerable groups in research.

VERSION 1 - AUTHOR RESPONSE

Reviewer: 1

Dr. S Cusworth Walker, University of Washington School of Medicine

Comments to the Author:

Thank you for the opportunity to review this study protocol regarding a participatory, research setting priority project with multisector, diverse constituents. The protocol was well-written and organized.

Introduction. The introduction provides well-justified arguments and citations regarding the value of focusing on UK coastal areas for mental health research and practice/policy intervention. The authors note the value of addressing disparities in research, but could also include more examples and citations of how community-engaged research leads to public impact. For example, the authors could reference the literature on Research Practice Partnerships, or community participatory research resulting in collaborative policy advocacy between researchers and community members. The intended or hypothesized link implicit in the researchers' laudable effort could be more explicit.

We thank the reviewer for their kind comments and feedback. We have now acknowledged and added the importance and relevance of community-based participatory research approach in our in our work, and have revised the last paragraph of the introduction as follows:

Addressing disparities in mental health research is a key government objective. However, for research to have a real impact and make a difference in people's lives, it needs to be developed and prioritised with key stakeholders and those whose lives will be most affected by the research. Informed by a community-based participatory research approach [41], our partnership with those directly affected by and knowledgeable of the local issues that affect mental health means that our research has real potential to influence the implementation of evidence and evidence-based interventions into practice and policy [42]. This is the key premise underpinning this Norfolk-Suffolk Mental Health Development Award (MHDA) project.

Methods. The methods were clearly described and included appropriate procedures for the researchers' aims, including multiple methods of capturing feedback from community members as part of initial prioritization. The researchers will want to take care that participation in in-person ranking exercises attempts to include those who may not routinely volunteer for those types of activities.

Thank you for this helpful suggestion. We have included the following to phased 4 – "Prioritisation workshop" recruitment strategy:

We will adapt our recruitment strategy to purposively target groups often underrepresented in research.

Aims. The phases of the study proceed logically and thoughtfully. Again, the researchers might consider being more explicit in how research priorities are expected to achieve practical changes as this may influence the types of capacity needed for the ongoing collaboration.

We have addressed this point in relation to the reviewer's earlier feedback on the introduction.

Congratulations to the authors on an exciting venture.

Thank you!

Reviewer: 2

Mrs. Patrice Whitehorne-Smith, Curtin University Faculty of Health Sciences

Comments to the Author:

This is a well designed and detailed protocol. It appears to be a highly relevant study. The methodology and strategies for inclusion of all critical stakeholders creates the foundation for a highly impactful and actionable body of research. I have three minor suggestions:

1. The introduction could be strengthened by bringing in literature related to the social determinants of mental health. Although some of these factors are alluded to, clearly demonstrating the factors you have identified within that framework would further justify the need for the inclusion of diverse stakeholders in the study and the intersectionality implied in your approach.

2. The introduction highlights an influx of immigrants within Norfolk and Suffolk, however your methods does not include an specific language based exclusion criteria. Will fluency in English be a consideration for participation in the study?

Thank you. This is an important observation. We can confirm that our participants need to be fluent in English language to take park. We have added this to the manuscript.

3. Phase 2b-in person survey and focus group discussions- although it was stated that focus groups will be conducted where participants are based, it is unclear if the travel costs to the location for the purpose of the focus group will be covered in this phase. Travel expenses can be a major deterrent to the participation of marginalised or vulnerable groups in research.

Thank you for this important point. Although, we stated in the Ethics section that travel costs will be covered. We agree with the reviewer that this could be made clearer in Phase 2b. As such, we have added this.
